# Laparoscopic assessment and transvaginal reparation of post-coital vaginal cuff dehiscence with bowel evisceration: a case report

**DOI:** 10.11604/pamj.2020.35.118.2136

**Published:** 2020-04-14

**Authors:** Feras Sendy, Luisa De Simone, Maël Albaut, Antonin Lambert, Erdogan Nohuz

**Affiliations:** 1 Department of Obstetrics and Gynecology, Thiers Hospital, Route du Fau, 63300 Thiers, France; 2 Université Clermont Auvergne, Faculty of Medicine, Place Henri Dunant, 63001 Clermont Ferrand, France; 3 Departement of Obstetrics and Gynecology, Hôpital Femme - Mère - Enfant (HFME), Hospices civils de Lyon, CHU de Lyon, 59 boulevard Pinel, 69000 Lyon, France

**Keywords:** Vaginal cuff dehiscence, laparoscopy, acute pelvic pain, total hysterectomy

## Abstract

Vaginal cuff dehiscence (VCD) is a rare postoperative complication of total hysterectomy. Presenting symptom is acute pelvic or abdominal pain accompanied by nausea and vomiting. Immediate recognition and surgical repair are crucial for successful management. A 40-year-old para 1+0 presented with complaints of pelvic pain associated with sexual activity, three months after a total laparoscopic hysterectomy. Speculum examination revealed the presence of bowel into the vagina. Diagnostic laparoscopic assessment combined with VCD repair through the transvaginal route. The occurrence of VCD after laparoscopic hysterectomy has been linked to overuse of electrocautery, prolonged inflammatory response and suturing methods. Laparoscopic, abdominal and vaginal approaches are the routes for repairing VCD. However, it depends on the clinical presentation and surgeon expertise. Careful history, and physical examination are vital factors in guiding clinicians to diagnose and treat VCD. Nevertheless, an ideal modality remains variable to each case.

## Introduction

Vaginal cuff dehiscence (VCD) is defined as a full or partial separation of the anterior and posterior edges of the vaginal cuff that was previously closed. This uncommon but potentially life-threatening complication occurring after total hysterectomy causes then a direct connection between the peritoneal cavity and the vagina. It can be accompanied with protrusion of abdominal or pelvic contents, through the vagina, causing a wide range of signs and symptoms from minimal vaginal discharge to profuse bleeding and intestinal evisceration leading to bowel ischemia and infection that requires immediate action. Presenting symptoms include acute pelvic or abdominal pain, vaginal bleeding, watery discharge, nausea and vomiting [1]. Here in, we report a case of VCD following total laparoscopic hysterectomy successfully repaired by vaginal route after immediate exploratory laparoscopy.

## Patient and observation

**Figure 1:**
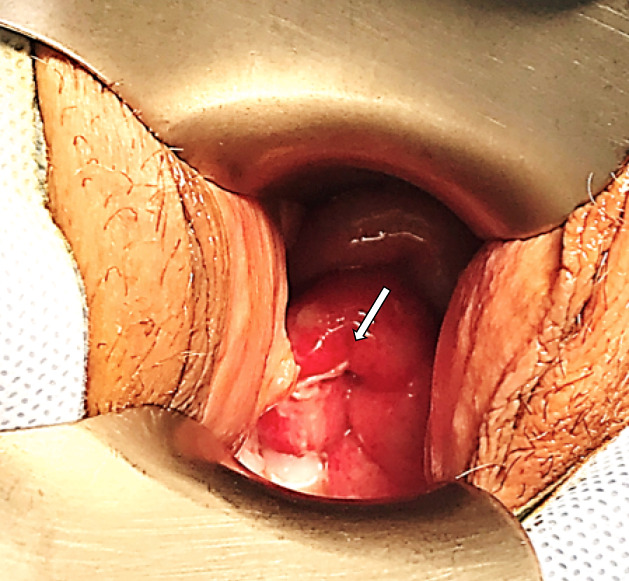
Pre-operative image of vaginal cuff dehiscence with bowel evisceration (arrow)

A 40-year-old woman presented to our hospital emergency department three months after she underwent a total laparoscopic hysterectomy (TLH) and bilateral salpingectomy for abnormal uterine bleeding unresponsive to medical treatment. During this initial surgery performed in another hospital, two endometriotic nodules of the uterosacral ligaments had also been resected. The vaginal cuff had been incised using monopolar electrocautery and closed vaginally using 0-Monocryl^®^ (Ethicon Inc. Somerville, NJ) interrupted sutures laparoscopically. Histological examination revealed adenomyotic uterus and confirmed the endometriotic origin of the nodules. The postoperative follow-up at one month was unremarkable with a properly healed vagina. The patient was consulted three months after her operation with a complaint of acute pelvic pain that was followed by nausea and vomiting. Before the onset of symptoms, the patient had sexual intercourse for the first time since her surgery. The husband revealed that during sexual intercourse, he suddenly did not have the same sensation of perception of the vaginal bottom. Physical examination showed a tense abdomen, particularly in the right lower quadrant with an overall score of pain estimated by the patient at 9/10. Speculum examination exposed small bowel free from necrosis protruding through the vaginal wall with the presence of peritoneal fluid (Figure 1).

**Figure 2:**
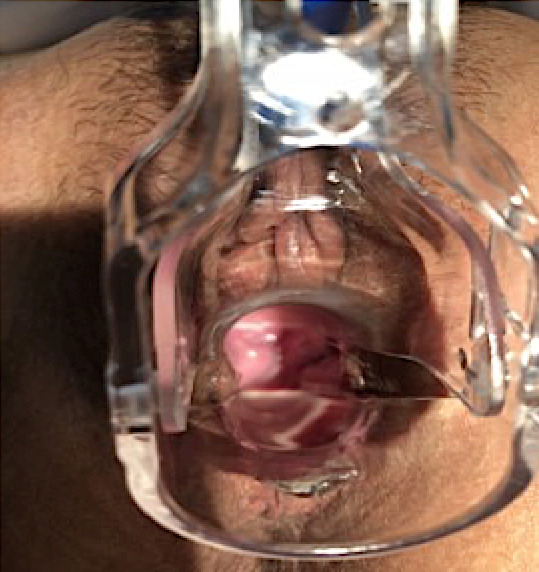
Post-operative image of a healed vaginal cuff dehiscence

Conversely, no vaginal bleeding or foul-smelling discharge was observed. The bowel was kept moist and warm with gauze. Preoperative laboratory workup was performed and showed a hemoglobin level of 13.9 and a platelet count of 271 with leukocytosis 11.9, a standard C reactive protein, urinalysis, and culture. These results were obtained postoperatively. After informed consent was acquired, the patient was taken to the operating theatre for urgent exploratory laparoscopy and underwent an inspection of the entire bowel, which was free of necrosis and adhesions to surrounding structures. Reduction of bowel evisceration into the abdomen and peritoneal wash via laparoscopy were followed by closure of a 5cm vaginal wall dehiscence by transvaginal route, after having resected the scarred and devitalized vaginal tissue at the margins to refresh it. To do this, an interrupted suture using an absorbable 0-Vicryl^®^ (Ethicon Inc., Somerville, NJ) was performed. The patient was discharged on a postoperative day 2 with an uncomplicated hospital course. Histological analysis of the resected vaginal tissue revealed no particular lesions other than inflammatory granuloma lacking specificity and malignancy. At her 4-months follow up, a well-healed vaginal cuff was palpated (Figure 2), the vagina had good caliber and length and sexual activity was resumed without complications.

## Discussion

Currently, minimally invasive surgeries are the preferred modality over abdominal surgery due to less pain, blood loss, smaller incisions, days of hospitalization, and rapid recovery [2]. Evidence demonstrates that for hysterectomy, vaginal route when feasible is associated with similar outcomes to the laparoscopic or abdominal route. Nevertheless, despite the finding that the vaginal route is associated with lower cost, total laparoscopic and robotic hysterectomy increase sensibly in practice [3]. However, the amplified risk of cuff dehiscence after laparoscopic and robotic hysterectomies compared with alternative approaches is recognized. Indeed, the incidence of VCD after hysterectomy was reported to be highest in robotic (1.64%) [4] followed by laparoscopy (0.64% - 1.35%) [4] respectively. This risk should thus be discussed with patients, about possible symptoms of post-operative cuff dehiscence mainly.

A literature review by Hur *et al.* (2016) [5] found that risk factors for VCD manifestation consist of vaginal atrophy, age, chronic steroid use, poor surgical technique, early resumption of coital activity, postoperative infections, postoperative hematoma, radiation therapy, foreign objects and increased intra-abdominal pressure (obesity, chronic cough, straining with defecation). Horgan and Gorey (1992) [6] reported that the color and peristalsis of the bowel might not affirm bowel survival. Thus, variables such as bowel evisceration, surgeon experience and stability of the patient could play an important role in choosing the suitable surgical approach for a VCD. In significance to the pros and cons of each surgical modality in line with the clinical presentation of our patient, we concluded that the modality of choice for the abdominopelvic cavity exploration was laparoscopy. Probable causes of VCD after TLH could be attributed to a prolonged inflammatory phase that could affect the reparation process [7], excessive use of electrocautery, suturing modalities and wound infection [8]. Another arguably cause for the rise in incidence could be the augmentation of utilization of minimally invasive procedures, as it is becoming the efficient mode of practice in the 21^st^ century. Numerous surgical modalities have been proposed to decrease the risk of VCD associated with endoscopic hysterectomy. They include the use of monopolar current on cutting mode, an accomplishment of cuff hemostasis with sutures rather than electrocoagulation, and the use of a two-layer cuff closure [9].

Reparation of VCD can be performed via abdominal, transvaginal, laparoscopic and robotic approaches. Urgent laparotomy was historically recommended to permit a complete bowel inspection. The appropriate modality of treatment depends on a variety of factors including patient presentation and stability, surgeon experience and availability of equipment. Therefore, careful history taking and clinical examination are key factors in diagnosing and treating vaginal cuff dehiscence. Recently, successful outcomes using a combined vaginal and laparoscopic approach have been reported. However, to our knowledge, only a few cases (a dozen) using this double approach have been reported. Although in this case we have not closed the vaginal dehiscence through a laparoscopic route. However, we were able to merge both the laparoscopic and transvaginal modalities. The potency of observation, surveillance and confirmation of closure via laparoscopy were imminent. Transvaginal closure appears to be the best remedy for improving knot integrity. The vaginal route was used to close the dehiscence in consideration of the feasibility to follow the integrity of the suture line, the disappearance of the absorbable thread, the exteriorization of the stitches out of the peritoneal cavity and the healing process via speculum examination at follow up visits.

Limited reports in the literature demonstrate the use of both modalities together in VCD; this emphasizes the value of the approach and possibly its application whenever feasible. The risk of dehiscence seems influenced mainly by the scope and complexity of the surgical procedure [10]. Moreover, improved surgeon experience can help reduce the incidence of VCD. In this case, the resection of two uterosacral ligaments endometriotic nodules can represent a risk factor of VCD due to the size of resected tissues which is a source of the fragility of the vaginal cuff. Literature is discordant concerning specific comorbidities (tobacco use or diabetes) concerning poor wound healing while. Recurrent VCD have been described and the time to its occurrence is variable, ranging from a few days to more than a year. Accordingly, the patient's information and education thus remain fundamental. It should also be remembered that partial resection of the vaginal stump dehiscence, before its suture, remains a necessity in order to restore a well-vascularized tissue and improve healing. Moreover, patients with VCD demonstrate significantly higher levels of acute and chronic inflammatory cells suggesting that a prolonged inflammatory phase may be delaying normal progression to reparation in these patients [8]. In this case report, histological analysis corroborates this physiopathological factor of VCD.

## Conclusion

Vaginal intercourse is the precipitating event of acute pelvic pain and occurring in a patient with recent history of total hysterectomy should alert physicians of VCD as a differential diagnosis. In determination, laparoscopy-assisted vaginal cuff suturing for vaginal cuff dehiscence after TLH. The coincidental of bowel injury or peritonitis ought be considered in case of vaginal cuff dehiscence, and diagnosis and surgical repair are the key factors for successful management.

## Competing interests

The authors declare no competing interests.
